# Differential Effects of Visual-Acoustic Biofeedback Intervention for Residual Speech Errors

**DOI:** 10.3389/fnhum.2016.00567

**Published:** 2016-11-11

**Authors:** Tara McAllister Byun, Heather Campbell

**Affiliations:** Department of Communicative Sciences and Disorders, Steinhardt School of Culture, Education, and Human Development, New York University, New YorkNY, USA

**Keywords:** biofeedback intervention, residual speech errors, articulation disorders, speech sound disorders, single-subject design, mixed-effects regression

## Abstract

Recent evidence suggests that the incorporation of visual biofeedback technologies may enhance response to treatment in individuals with residual speech errors. However, there is a need for controlled research systematically comparing biofeedback versus non-biofeedback intervention approaches. This study implemented a single-subject experimental design with a crossover component to investigate the relative efficacy of visual-acoustic biofeedback and traditional articulatory treatment for residual rhotic errors. Eleven child/adolescent participants received ten sessions of visual-acoustic biofeedback and 10 sessions of traditional treatment, with the order of biofeedback and traditional phases counterbalanced across participants. Probe measures eliciting untreated rhotic words were administered in at least three sessions prior to the start of treatment (baseline), between the two treatment phases (midpoint), and after treatment ended (maintenance), as well as before and after each treatment session. Perceptual accuracy of rhotic production was assessed by outside listeners in a blinded, randomized fashion. Results were analyzed using a combination of visual inspection of treatment trajectories, individual effect sizes, and logistic mixed-effects regression. Effect sizes and visual inspection revealed that participants could be divided into categories of strong responders (*n* = 4), mixed/moderate responders (*n* = 3), and non-responders (*n* = 4). Individual results did not reveal a reliable pattern of stronger performance in biofeedback versus traditional blocks, or vice versa. Moreover, biofeedback versus traditional treatment was not a significant predictor of accuracy in the logistic mixed-effects model examining all within-treatment word probes. However, the interaction between treatment condition and treatment order was significant: biofeedback was more effective than traditional treatment in the first phase of treatment, and traditional treatment was more effective than biofeedback in the second phase. This is consistent with existing theory and data suggesting that detailed knowledge of performance feedback is most effective in the early stages of motor learning. Further research is needed to confirm that an initial phase of biofeedback has a facilitative effect, and to determine the optimal duration of biofeedback treatment. In addition, there is a strong need for correlational studies to examine which individuals with residual speech errors are most likely to respond to treatment.

## Introduction

Visual biofeedback utilizes instrumentation to provide a real-time display of some physiological function or behavior, intended to help learners gain conscious control over these functions (Davis and Drichta, 1980; Volin, 1998). When biofeedback is used in speech intervention, a visual model representing a correctly produced speech target can be displayed above or next to the real-time display. A speech-language pathologist (SLP) typically provides cues to help the learner shape his/her output into a closer match for the model. A common approach provides a real-time image of the articulators using technologies such as electromagnetic articulography (e.g., Katz et al., 2010), ultrasound (e.g., Adler-Bock et al., 2007; Preston et al., 2013, 2014, 2016; McAllister Byun et al., 2014b), or electropalatography (e.g., Gibbon et al., 1999). An alternative approach is visual-acoustic biofeedback, in which learners view a dynamic representation of the formants or resonant frequencies of the vocal tract (Shuster et al., 1992, 1995; McAllister Byun and Hitchcock, 2012; McAllister Byun et al., 2016b).

The rationale for incorporating biofeedback into treatment is grounded in the literature investigating principles of motor learning, or PML (Maas et al., 2008; Bislick et al., 2012). The PML framework recognizes a distinction between the initial acquisition of a motor skill, as revealed by the learner’s performance within the practice setting, versus subsequent retention and generalization of learned skills to other contexts. Different parameters of practice, such as the intensity of treatment and the frequency and type of feedback provided, have been argued to have differential impacts on the acquisition versus generalization of motor skills (Maas et al., 2008). Visual biofeedback provides a highly detailed type of qualitative or *knowledge of performance (KP)* feedback. KP feedback is thought to facilitate the acquisition of new motor skills by helping the learner understand how to achieve an unfamiliar target (Maas et al., 2008; Preston et al., 2013). However, it has been argued that KP feedback can have a neutral or even negative impact on long-term retention and generalization of motor skills, particularly if learners become overly dependent on the detailed external feedback (Hodges and Franks, 2001; Maas et al., 2008). Thus, it is possible that any advantage for biofeedback over non-biofeedback treatment approaches may be specific to the early stages of treatment. In keeping with this, previous biofeedback treatment research has reported that changes in speech production are readily induced within the treatment setting for most participants, but these gains do not automatically generalize to a setting in which biofeedback is not available (Gibbon and Paterson, 2006; McAllister Byun and Hitchcock, 2012).

It should be noted that there are other possible explanations for how biofeedback has its effect. Studies of non-speech motor learning have reported that both acquisition and generalization of new motor skills are facilitated when the learner adopts an external direction of attentional focus, e.g., attending to an implement or visual display instead of his/her own body movements. Biofeedback encourages the learner to adopt an external direction of focus during speech-motor tasks, which could potentially account for improved learning in biofeedback relative to non-biofeedback treatment conditions (see discussion in McAllister Byun et al., 2016b).

At the present time, the efficacy of visual biofeedback interventions for speech is supported by case studies and/or single-subject experimental designs, which correspond with Phases I and II in Robey’s (2004) 5-phase model of clinical research. The following section will provide more detailed evidence for the efficacy of a specific type of biofeedback (visual-acoustic biofeedback) in a specific treatment context (residual rhotic errors). At this point we merely note that previous research has acknowledged the need for larger and more systematic studies to measure the efficacy of biofeedback relative to more traditional speech interventions. In addition, understanding *why* biofeedback has its effect will be important in determining how it can optimally be incorporated into a treatment program structured in accordance with principles of motor learning.

## Visual-Acoustic Biofeedback for Residual Rhotic Errors

Speech sound disorders can limit children’s participation in social and academic activities, which may exert a negative impact on psycho-emotional development (McCormack et al., 2009; Hitchcock et al., 2015). For most children, developmental speech errors resolve by 8 or 9 years of age; errors that persist past this point can be termed residual speech errors, or RSEs (Shriberg et al., 1994). RSEs are perceived as a particular challenge by pediatric SLPs, who report that a subset of children with RSEs remain unresponsive to traditional treatment approaches (Ruscello, 1995). One of the most common residual errors is misarticulation of the North American English rhotic /ɹ/. In the population at large, rhotic production does not reach mastery level until approximately 8 years of age (Smit et al., 1990), making it one of the latest-emerging speech sounds in English. Children’s difficulty acquiring correct /ɹ/ production is believed to be partially explained by the motoric complexity required to produce the sound (Adler-Bock et al., 2007).^1^ Acoustically, /ɹ/ is differentiated from other sonorants by a particularly low frequency of the third formant, F3 (Delattre and Freeman, 1968; Hagiwara, 1995), which combines with a relatively high second formant frequency (F2) to yield a small F3–F2 distance (Boyce and Espy-Wilson, 1997). This paper follows a clinically common convention in differentiating between consonantal and vocalic categories of rhotics. The term consonantal rhotic is used for /ɹ/ in syllable onset position. Vocalic rhotics will be treated as a broader category including both syllabic variants (e.g., *fur*, /fɝ/; *water*, /wɔɾɚ/) and the post-vocalic variant, which can be transcribed as the offglide of a rhotic diphthong (e.g., *dare*, /dɛɚ/; *deer*, /dɪɚ/; *door*, /dɔɚ/). Although this distinction is supported by acoustic evidence (Shriberg et al., 2001; McGowan et al., 2004) as well as evidence from treatment studies (McAllister Byun and Hitchcock, 2012; Preston et al., 2013), there is still debate over the most accurate characterization and subcategorization of the positional allophones of the English rhotic (Lockenvitz et al., 2015).

**FIGURE 1:**
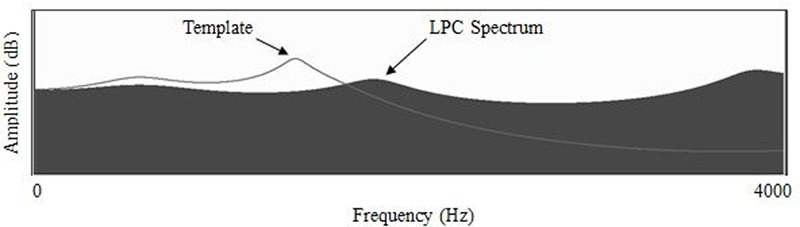
**Formant frequencies represented as peaks of an LPC spectral display, with line representing an accurate rhotic target.** From McAllister Byun and Hitchcock (2012); used with permission.

Treatment for residual rhotic errors commonly follows a traditional articulatory approach (e.g., Van Riper and Erickson, 1996) in which the clinician combines auditory models with verbal cues intended to encourage a more appropriate lingual posture. Visual-acoustic biofeedback is a treatment approach that shifts the focus away from articulator placement cues and instead encourages the learner to match a visually displayed acoustic target. The current study provided treatment for residual rhotic errors using the real-time LPC function of the Sona-Match program within the KayPentax Computerized Speech Lab (CSL) software suite. **Figure 1** depicts an example of an LPC display used for visual biofeedback. The solid wave-like shape in **Figure 1** is the real-time LPC spectrum, which tracks the locations of speaker’s formant frequencies from instant to instant. The superimposed line is the acoustic template representing a model speaker’s production of a perceptually accurate rhotic.^2^ Note that in the template in **Figure 1**, the second and third formants appear to have merged into a single peak; this is not uncommon due to the very small distance between F3 and F2 in highly rhotic productions of /ɹ/ (Boyce and Espy-Wilson, 1997; Flipsen et al., 2001; Shriberg et al., 2001).

Visual biofeedback intervention has shown promise as a means to establish perceptually accurate rhotic production in children whose residual rhotic errors have not responded to previous forms of treatment. Two early case studies found spectrographic biofeedback to be effective in eliciting accurate /ɹ/ productions from three adolescents with residual rhotic errors; these gains were also observed to generalize beyond lab-based biofeedback practice (Shuster et al., 1992, 1995). In a quasi-experimental study (McAllister Byun and Hitchcock, 2012), 11 children received a phase of traditional treatment followed by a phase of acoustic biofeedback using an LPC spectrum. Word-level generalization probes collected after the initial phase of traditional treatment revealed no significant change in accuracy relative to baseline sessions, but probes collected after the biofeedback phase showed significant gains in perceptually rated accuracy that were corroborated by acoustic measures. Another single-subject experimental study (McAllister Byun et al., 2016b) investigated the impact of 8 weeks of LPC-based biofeedback treatment with nine children with RSE who had previously received at least 5 months of traditional therapy without success. Six of the children demonstrated improved production of at least one treated target in generalization probes elicited after the end of treatment, and a mixed-effects logistic model indicated that rhotic productions in post-treatment maintenance sessions were significantly more likely to be rated accurate by blinded listeners than the same words produced at baseline.

One limitation of the literature reviewed above is the lack of true experimental studies exploring the relative impact of visual-acoustic biofeedback versus traditional approaches to intervention. The quasi-experimental study in McAllister Byun and Hitchcock (2012) included both forms of treatment, but the design did not preclude the possibility that gains observed during the biofeedback phase could represent a late-emerging response to the initial phase of traditional treatment. In addition, although McAllister Byun et al. (2016b) measured the efficacy of visual-acoustic biofeedback using a single-subject experimental design, that study did not include a traditional treatment comparison condition. The current single-subject experimental study was designed to fill the need for a well-controlled comparison of traditional and biofeedback intervention approaches for RSE. Participants received one 10-session phase of traditional treatment and one 10-session phase of biofeedback treatment, counterbalanced in order across participants. It was hypothesized that visual inspection and individual effect sizes would reveal moderate to strong evidence of improvement on generalization probes for most or all phases of treatment. It was also hypothesized that across-subjects comparison using mixed-effects logistic regression would reveal a significant advantage for biofeedback over traditional treatment.

## Materials and Methods

### Participants

The present study enrolled eleven native speakers of American English who ranged in age from 9;3 to 15;10 (mean age 11;3, SD 25 months). Seven participants were male, and four were female, a ratio consistent with previous descriptions of the gender distribution of residual speech errors (Shriberg, 2010). Participants were recruited through flyers distributed to schools and community centers in the New York City metropolitan area. Participants were required to satisfy two sets of criteria for inclusion in the study. The first set pertained to the child’s developmental history and language exposure, as determined via parent report on a questionnaire. The second set of criteria pertained to the child’s performance on an initial evaluation of speech and language function. The study protocol was approved by the Institutional Review Board at New York University.

Participants were required to have native-level knowledge of English, but in light of the linguistically diverse nature of the urban population from which this sample was drawn, exposure to languages other than English was not an exclusionary criterion. Three participants (Bryce, Landon, and Cooper) were described as early sequential bilinguals who heard English at school and English and another language (Arabic, Hebrew, and Mandarin, respectively) in the home. The parent questionnaire also asked what dialects of English were spoken in the home, with a question specifically probing whether the child was exposed to a dialect with “r-deletion,” such as British English or a traditional New York dialect. The current sample included no children for whom exposure to an r-deleting dialect appeared to be a factor contributing to rhotic misarticulation. The questionnaire additionally asked for an estimate of the duration of previous treatment that the child had received targeting rhotic errors. The reported treatment durations ranged from 6 months to 4.5 years (median duration 2;0 years). While enrolled in the study, participants were not permitted to receive other intervention for rhotics. Finally, parents were asked to report whether their child had any history of developmental or neurobehavioral disorder. No comorbid diagnoses (e.g., ADHD, language-based learning disability) were reported, although an early history of developmental delay was reported for one participant. **Table 1** provides a summary of speech and language history information for all participants. Names are pseudonyms.

**Table 1:** Participant background data, per parent report.

Pseudonym	Gender	Age at enrollment	Duration of previous treatment targeting rhotics	Other previous or current speech targets (per parent report)
Aiden	M	10;2	No response	/g, k/, /l/
Bryce	M	9;5	4.5 years (school)	No response
Cooper^∗^	M	10;1	3 years (school)	/t∫, ∫ /, /p, b/, /t/
Erica	F	9;3	1 year (private)	/t, d/, /t∫, ∫ /, /𝜃/
Ella	F	13;8	0.5 years (private)	None
Gregory	M	9;10	0.5 years (school)	None
Holly	F	11;4	4 years (school and private)	/s, ∫ /
Harper	F	11;9	1.5 years (school and private)	None
Jason	M	15;10	3.5 years (private)	None
Landon	M	9;6	2 years (school)	/l, w/
Mason	M	12;10	No response	No response

*^∗^Indicates parent report of developmental delay.*

**Table 2** summarizes the results of the initial evaluation that furnished the second set of inclusionary criteria. All participants were required to pass a pure-tone hearing screening at 500, 1000, 2000, and 4000 Hz at 20 dB HL. They also showed no abnormal structure and/or function of the speech and hearing mechanism, as determined by a certified SLP using an adapted version of Shipley and McAfee’s (2008) checklist. Receptive language was required to fall within normal limits, as indicated by a score within one standard deviation of the mean for the child’s age on the “Auditory Comprehension” subtest of the *Test of Auditory Processing Skills-3rd Edition* (*TAPS-3*, Martin and Brownell, 2005). Other than rhotic errors, enrollees were required to produce no more than three sounds in error on the Goldman-Fristoe Test of Articulation-2nd Edition (GFTA-2, Goldman and Fristoe, 2000).^3^ Some participants did exhibit residual errors on other late-developing sounds, including /s/ and /l/.

**Table 2:** Participant evaluation results.

Pseudonym	TAPS-3 Scaled Score (percentile)	Non-rhotic error sounds (GFTA-2)	Rhotic word probe: Number fully correct out of 38 vocalic items	Rhotic word probe: Number fully correct out of 25 consonantal items
Aiden	14 (91)	/k/, /t∫ / (minor distortion)	0	0
Bryce	14 (91)	/s, z/ (minor distortion)	0	0
Cooper	11 (63)	/l/, /s, z/, /𝜃/	0	0
Erica	12 (75)	/s, z/, /∫ /, /t∫, dʒ/	0	0
Ella	19 (98)	none	0	1
Gregory	11 (63)	none	0	0
Holly	11 (63)	/s, z/, /𝜃/	0	6
Harper	8 (25)	none	0	0
Jason	10 (50)	none	0	0
Landon	8 (25)	/l/	0	0
Mason	13 (84)	none	2	0

To enroll a sample that was relatively homogenous with respect to rhotic production accuracy prior to the onset of treatment, all participants were required to exhibit no more than 30% fully correct production of rhotics at the word level on a 70-item probe. To this end, all 70 productions were rated as either correct or incorrect by two experienced listeners who followed a consensus procedure (Shriberg et al., 1984) and maintained the strict criterion that all distorted/intermediate productions should be classified as incorrect. **Table 2** reports individual percentages of items rated correct on this word probe, subdivided into vocalic and consonantal variants. Elsewhere in this paper, participants’ rhotic production accuracy will be estimated using perceptual ratings obtained from naïve listeners, who tend to be more lenient in their ratings of children’s rhotic sounds than trained experts (McAllister Byun et al., 2015).

### Instruments and Procedures

The present study used a multiple-baseline across-subjects design with a crossover component to document the efficacy of visual-acoustic biofeedback intervention relative to a comparison condition involving traditional articulatory treatment. For the multiple baseline component, participants were randomly assigned to complete rhotic production probes in three, four, or five sessions prior to the beginning of treatment. For the crossover component, they were randomly assigned to begin treatment in either a biofeedback or a traditional treatment condition, with the type of treatment switching halfway through the study. As a measure of long-term progress over the course of treatment, participants’ rhotic production accuracy was probed in three midpoint sessions at the point of switch between the two types of therapy, as well as in three maintenance sessions following the final treatment session. All baseline, midpoint, and maintenance sessions featured the same 50-word probe containing rhotics in various phonetic contexts, administered in a randomized order (see complete list in Appendix A). As a measure of short-term change within treatment, a fixed 25-word subset probe was presented in randomized order at the beginning and end of each of the 20 treatment sessions. Probe words were not used as targets in treatment.

This study was structured to meet What Works Clearinghouse (WWC) standards for single-subject experimental design (Kratochwill et al., 2010). The independent variable, application of traditional and biofeedback treatment, was systematically manipulated by the experimenters. The outcome variable (perceptually rated accuracy of rhotic production) was tracked over time by multiple raters whose interrater agreement was established using psychometrically accepted criteria. The inclusion of three different baseline durations (three, four, and five sessions) meets the minimum criterion of three opportunities to demonstrate an intervention effect at three different points in time. The use of a minimum of three (rather than five) points of data collection in each phase of the study meets WWC standards “with reservations,” rather than “in full.”

### Intervention Characteristics

The second author, a licensed and certified SLP, delivered treatment in all sessions for all participants. Extended introductory instructions were provided at the start of the first two sessions of each treatment type. In the traditional treatment condition, the clinician presented illustrations and verbal descriptions to familiarize participants with articulatory configurations that can facilitate perceptually accurate production of rhotics. Based on previous evidence, cues targeted three specific components of lingual placement for rhotic production: retraction of the tongue root (Adler-Bock et al., 2007; Klein et al., 2013), elevation of the lateral margins of the tongue blade/body (Bacsfalvi, 2010), and elevation of the anterior tongue. The clinician did not specify a particular configuration of the anterior tongue (e.g., retroflex versus bunched), since previous research has suggested that learners may benefit from opportunities to explore different configurations to find the best fit for their individual vocal tract (Klein et al., 2013; McAllister Byun et al., 2014b). In the biofeedback treatment condition, the initial sessions served to familiarize participants with the real-time LPC spectrum. They were given opportunities to match formant targets using sounds that they could produce accurately (e.g., /i/, /ɑ/). They were also familiarized with images representing the spectra of both correct and incorrect rhotic productions.

All 20 sessions included a 5-min interval of “free play,” a relatively unstructured period in which the clinician could guide the client through practice producing rhotics in various phonetic contexts. The cues provided during free play were consistent with the current phase of treatment (i.e., traditional or biofeedback). After the free play interval, the treatment period elicited 60 rhotic production trials in blocks of five. Each block was preceded by one verbal cue consistent with the current treatment condition. For example, in the traditional treatment condition, the participant might hear the cue, “Try to make the back part of your tongue go back, like for /ɑ/.” In the biofeedback treatment condition, cues could reference the biofeedback display (e.g., “Focus on making the third bump move over”); articulatory cues were not provided. After each block, the clinician provided summary feedback by indicating which of the five trials she perceived to be the most accurate.

It has been suggested (Hitchcock and McAllister Byun, 2014) that generalization of gains in biofeedback treatment for RSE could be enhanced by incorporating principles from a “challenge point framework” (Guadagnoli and Lee, 2004; Rvachew and Brosseau-Lapré, 2012). The challenge point framework is described as an approach in which the participant is held at a level of difficulty that is neither too hard nor too easy, which is thought to maximize opportunities for learning (Rvachew and Brosseau-Lapré, 2012). During treatment sessions in the current study, adaptive adjustment of practice difficulty was implemented via custom software (Challenge-/r/, McAllister Byun et al., 2014a) that presents stimulus words and records accuracy ratings entered by the treating clinician. Following each set of ten trials, the program calculates the participant’s accuracy and makes corresponding adjustments to several parameters, such as the level of support provided or the difficulty of the stimulus items. When a participant demonstrates at least 80% accuracy over ten trials, the program advances one parameter to increase the difficulty level; if the score over ten trials drops below 50%, the program moves back one parameter to decrease difficulty. Parameters are adjusted on a rotating basis so that as accuracy increases, either the frequency of feedback is reduced (100%-50%-20%), clinician models are faded, or word shapes increase in complexity.

As the majority of studies agree that vocalic rhotics tend to be mastered earlier than consonantal /ɹ/ (Klein et al., 2013; Magloughlin, 2016), the current study limited initial treatment targets to vocalic variants. For each treatment session, a stratified subset of treatment words was selected, comprising equal proportions of the categories /ɑɚ/, /ɔɚ/, /ɛɚ/, /ɪɚ/, and /ɝ, ɚ/. A criterion was put in place for participants to advance to consonantal targets if they exceeded a predetermined threshold of performance, but no participant in the present study reached this criterion. Because no participant received treatment on consonantal /ɹ/, and little generalization to the untreated variant was observed, the discussion that follows will focus on words representing the treated category of vocalic rhotics.

#### Treatment Fidelity

Fidelity to the stated treatment protocol is an important consideration in intervention research (Kaderavek and Justice, 2010). Both the clinician and a student assistant consulted a detailed checklist throughout each session so that any deviations from protocol could be quickly detected and corrected. This online fidelity checking was particularly valuable as a means to prevent traditional articulatory cues from being provided during biofeedback treatment sessions. In addition, *post hoc* fidelity ratings were obtained for 2–3 sessions from each participant (31 total), balanced by treatment condition and point in the course of treatment. To assess fidelity, a research assistant who was not involved in treatment delivery completed a checklist to verify various aspects of the study design: (1) a cue preceded each block of five trials, (2) the cue was in alignment with the designated treatment condition (biofeedback/traditional); (3) each block consisted of exactly five trials; (4) feedback or other interruptions did not occur within a block; (5) summary feedback followed each block of five trials, when indicated.

### Measurement

Word-level productions elicited in all probe sessions were rated using Amazon Mechanical Turk (AMT), an online crowdsourcing platform that can facilitate the process of recruiting a large pool of participants to complete experimental tasks. Online crowdsourcing has come to represent a valuable resource for researchers in fields such as behavioral psychology (e.g., Paolacci et al., 2010) and linguistics (e.g., Sprouse, 2011). Even though this approach yields ratings that are inherently more variable than lab-based data, as the number of raters included in the crowdsourced pool increases, crowdsourced ratings begin to converge with those of expert raters (Ipeirotis et al., 2014). Numerous studies have reported that results obtained through AMT are comparable to those obtained in a lab-based setting (e.g., Paolacci et al., 2010; Sprouse, 2011; Crump et al., 2013). McAllister Byun et al. (2015) investigated the validity of crowdsourced data collection in the specific context of ratings of children’s productions of rhotic sounds. They found that binary ratings aggregated over 250 naïve listeners on AMT were highly correlated with binary ratings aggregated over 25 expert listeners (*r* = 0.92) and with an acoustic measure of rhoticity, F3-F2 distance (*r* = -0.79). Bootstrap analyses revealed that when nine or more AMT listeners were included in a subsample of raters, the aggregated AMT ratings converged with ratings aggregated over subsamples of three expert listeners, considered the “industry standard.” The accuracy of each token can be estimated by aggregating responses across raters using 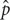
_correct_, which is the proportion of listeners who in a binary forced-choice task responded that the token was a “correct r sound” (McAllister Byun et al., 2016a).

**FIGURE 2:**
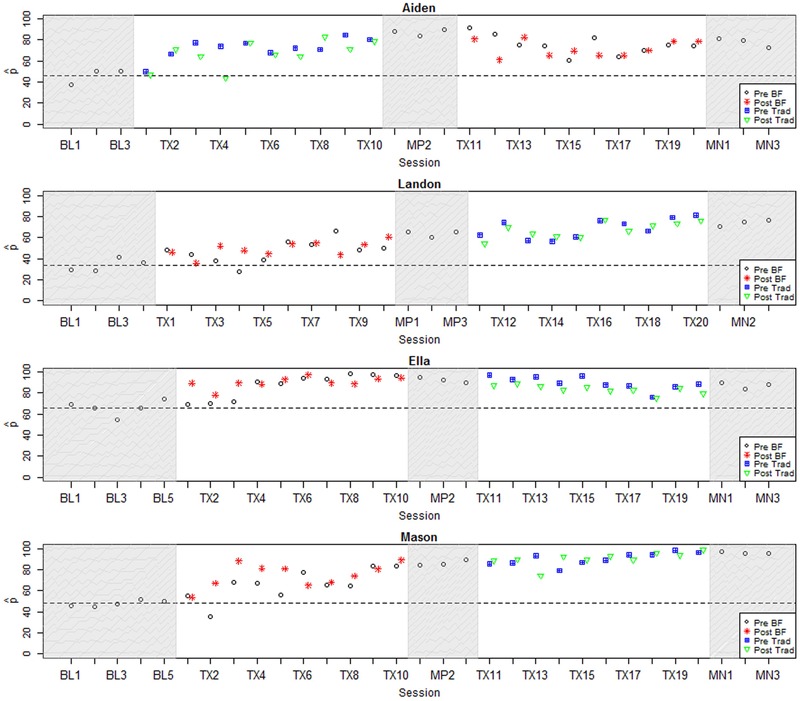
**Longitudinal plots of 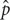
_correct_ for participants with large positive effect sizes.** Dashed line represents mean across baseline sessions. BL, Baseline; Tx, Treatment; MN, Maintenance; BF, Biofeedback; Trad, Traditional.

**FIGURE 3:**
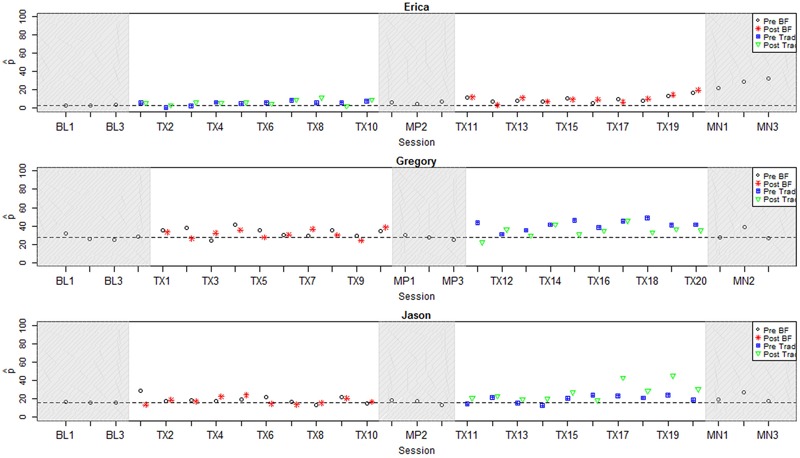
**Longitudinal plots of 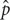
_correct_ for participants with small positive effect sizes.** Dashed line represents mean across baseline sessions. BL, Baseline; Tx, Treatment; MN, Maintenance; BF, Biofeedback; Trad, Traditional.

**FIGURE 4:**
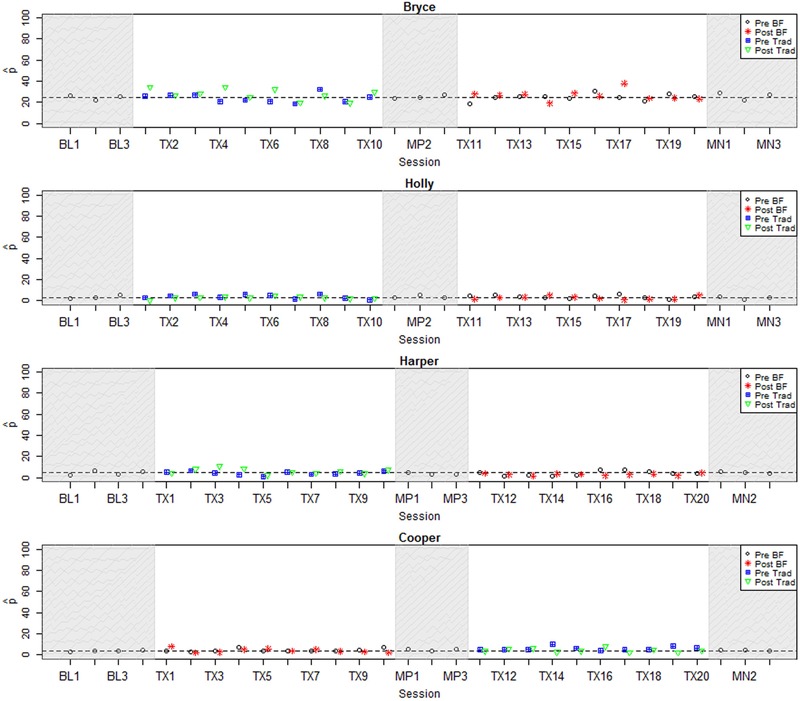
**Longitudinal plots of 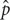
_correct_ for participants with null or negative effect size.** Dashed line represents mean across baseline sessions. BL, Baseline; Tx, Treatment; MN, Maintenance; BF, Biofeedback; Trad, Traditional.

In the current study, binary ratings of each speech token were collected from at least nine AMT listeners according to the protocol introduced in McAllister Byun et al. (2015); see Appendix B for additional detail on this protocol. For the plots in this paper (**Figures 2**–**4**), 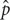
_correct_ was averaged across all vocalic targets within a session and converted to a percentage, i.e., the percentage of “correct r” ratings out of the total number of ratings for all items in a session.^4^ All participants had US-based IP addresses and, per self-report, were native speakers of English with no history of speech or hearing impairment. A total of 378 unique attempts were recorded, of which 126 were excluded for chance-level performance on catch trials and 21 were excluded for incomplete data. The final sample consisted of 226 responses. Users reported a mean age of 35.1 years (SD 9.7 years).^5^ Across the full set of included responses, interrater reliability was calculated to be 84.5%.

The Institutional Review Board at New York University approved the use of AMT to obtain speech ratings, and participants and their parents gave permission for recordings to be shared anonymously with outside listeners for the purpose of rating.

### Analyses

Following the conventional approach to the analysis of single-subject experimental data (Kratochwill et al., 2014), the results for each participant were examined for visual evidence of a treatment effect. Visual analysis is known to have limitations such as low interrater reliability (e.g., Brossart et al., 2006), and it has been suggested that the scientific credibility of single-subject experimental research could be enhanced through the addition of quantitative evidence such as effect sizes, hypothesis tests, and confidence intervals (Kratochwill and Levin, 2014). Hence, visual inspection, effect sizes, and a mixed-effects logistic regression model were utilized to explore treatment effects both within and across participants. By exploring the data through multiple analytical lenses, it should be possible to obtain an overall impression of participants’ response to treatment that is more robust than any single metric.

Because this study featured two phases of treatment, three effect sizes were calculated for each participant. The first effect size (*ES_Phase1_*) compared rhotic production accuracy in word probes from the pre-treatment baseline phase versus the midpoint phase, after the completion of the first phase of treatment. The second effect size (*ES_Phase2_*) computed the change in rhotic accuracy from the midpoint phase to the post-treatment maintenance phase. Finally, an overall effect size (*ES_All_*) compared performance in the baseline versus the maintenance phase. All effect sizes were calculated using 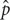
_correct_ pooled across all treated (i.e., vocalic) targets. Effect sizes were standardized using Busk and Serlin’s *d_2_* statistic (Beeson and Robey, 2006), which pools an individual’s standard deviation across the two phases being compared in order to reduce the number of cases in which an effect size cannot be calculated due to zero variance. Following Maas and Farinella (2012), an effect size of 1.0 (i.e., the difference between pre- and post-treatment means exceeds the pooled standard deviation) was adopted as the minimum *d_2_* that would be considered clinically relevant. Because *d_2_* can yield an inflated effect size estimate when variance is low, unstandardized effect sizes were also calculated and taken into consideration in the interpretation of each participant’s response to treatment.

The effect sizes discussed in the preceding paragraph reflect participants’ long-term or generalization gains in response to treatment. However, it is also possible for participants to make short-term gains that are evident on pre- and/or post-test probes during treatment, but may not be sustained into the midpoint or maintenance phases. To take these gains into consideration, a logistic mixed model (e.g., Rindskopf and Ferron, 2014) was fitted over all data points from pre- and post-treatment probes elicited during the two phases of treatment. For this analysis, we used an uncollapsed data set in which each data point was a single listener’s rating of a single token. The binary rating (correct/incorrect) served as the dependent variable. Random intercepts were included to reflect the fact that data points were nested within subjects, items, and raters. Fixed effects of treatment condition (biofeedback versus traditional) and order of treatment delivery (biofeedback-first versus traditional-first) were of primary interest relative to our theory-derived hypotheses; the time of probe administration (pre-treatment versus post-treatment) was also included. Individual subject properties that could potentially modulate response to treatment were additionally examined, including age in months, years of previous treatment targeting rhotics, and mean accuracy in the baseline phase based on blinded listener ratings (as reported in **Table 3**). Random slopes were examined as permitted by model convergence. Model selection was performed using likelihood ratio tests, and only those predictors, interactions, and random slopes that yielded a significant difference in likelihood relative to a minimally reduced model were retained.

**Table 3:** Individual effect sizes.

Pseudonym	Baseline mean 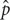 _correct_ (*SD*)	Midpoint mean 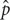 _correct_ (SD)	Maintenance mean 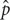 _correct_ (SD)	Phase 1 Effect Size (*ES_Phase1_*)	Phase 2 Effect Size (*ES_Phase2_*)	Overall Effect Size (*ES_all_*)
Aiden	46.03 (7.43)	87.31 (3.02)	78.01 (4.46)	7.28	-2.44	5.22
Bryce	24.5 (1.87)	25.33 (1.96)	26.10 (3.26)	0.44	0.29	0.60
Cooper	3.51 (0.49)	4.96 (0.98)	4.24 (0.41)	2.00	-0.96	1.60
Erica	3.25 (0.77)	6.27 (1.14)	27.91 (4.91)	3.12	6.07	7.01
Ella	66.04 (7.24)	92.47 (2.54)	87.08 (3.19)	4.34	-1.87	3.40
Gregory	27.54 (3.19)	27.64 (2.49)	31.11 (7.03)	0.03	0.66	0.70
Holly	2.98 (1.83)	3.41 (1.58)	2.55 (1.33)	0.25	-0.59	-0.27
Harper	4.50 (1.83)	3.70 (1.32)	4.90 (1.23)	-0.48	0.94	0.25
Jason	15.81 (0.35)	15.95 (2.92)	20.89 (5.07)	0.07	1.19	1.41
Landon	33.86 (6.04)	63.96 (2.96)	73.99 (3.41)	5.98	3.14	7.79
Mason	47.94 (2.84)	85.98 (2.78)	96.12 (0.74)	13.51	4.98	20.45

## Results

### Fidelity

As indicated above, recordings of 15% of all treatment sessions were reviewed to verify fidelity to the stated treatment protocol. The results of these fidelity checks suggested that the clinician’s adherence to protocol in this study was very high. Verbal cues were uniformly judged to be consistent with the treatment condition designated for a session: student raters reported no cases of articulatory cues being provided during biofeedback treatment sessions. The most frequent deviation from protocol was the absence of a verbal model prior to a block of trials when a model should have been provided; this was reported in 6% of trials reviewed. Within-block interruptions, often to redirect the child to the task, were an occasional source of deviation from protocol, occurring in roughly 5% of sessions.

### Individual Results: Effect Sizes

Effect sizes representing change in 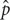
_correct_ for the treated variant, vocalic /ɹ/, are reported for all participants in **Table 3**. The first column shows participants’ mean 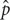
_correct_ in the baseline period, averaged across all vocalic /ɹ/ items from all baseline sessions. The second column shows the equivalent mean across the three midpoint sessions, and the third shows the three maintenance sessions. The next three columns report three standardized effect sizes: *ES_Phase1_* compares baseline versus midpoint scores, *ES_Phase2_* compares midpoint versus maintenance scores, and *ES_All_* compares baseline versus maintenance, reflecting overall gains across both phases of treatment. Participants are blocked by the order in which they received treatment (traditional-first or biofeedback-first). The effect sizes in **Table 3** show a wide range of variability in overall response to treatment across individuals. Averaging *ES_All_* values across all participants yields a mean of 4.38, suggesting that on average, participants’ response to the combined biofeedback and traditional treatment package was positive and exceeded the minimum value considered clinically significant. Individual patterns of response will be examined in detail in the next section.

### Individual Results: Visual Inspection

**Figures 2**–**4** represent each participant’s pattern of change in accuracy (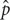
_correct_) over time, which can be visually inspected to corroborate the effect sizes reported in **Table 3**. The single-subject plots in **Figures 2**–**4** represent each child’s performance across the two treatment phases as well as baseline, midpoint, and maintenance probe stages. The y-axis represents 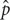
_correct_ aggregated across all vocalic /ɹ/ items within a session.^6^ Pre- and post-treatment probe measures are represented by different symbols (circles and stars in the biofeedback treatment condition and squares and triangles in the traditional treatment condition). The distance between pre- and post-treatment probes in a session thus provides an index of the participant’s progress during that session. Finally, a dashed horizontal line tracks the participant’s mean 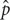
_correct_ from the baseline interval, so that subsequent scores can be compared to the baseline mean.

All participants were judged to demonstrate a sufficiently low level of baseline variability, defined as <10% mean session-to-session variability across the baseline phase. There were no outlier baseline sessions, defined as a session whose accuracy fell more than 2 standard deviations from the mean accuracy across the baseline phase for a given participant. Visual inspection raised questions of a possible rising trend during the baseline phase for two participants, Aiden and Ella. In Aiden’s case, the first data point was particularly low, while data points 2 and 3 show a higher but stable level of accuracy. Aiden’s performance on pre- and post-treatment probes in the first treatment session was virtually identical to the second and third baseline sessions, suggesting that these data points can be considered a stable and accurate reflection of his pre-treatment accuracy. In Ella’s case, the last three points of the baseline phase form a rising trend that terminates slightly above her mean accuracy across the baseline phase. However, once treatment was initiated, her accuracy in pre-treatment probes remained steady across the first three sessions, whereas post-treatment probe measures showed substantially increased accuracy. In subsequent sessions, both pre- and post-treatment probes showed elevated accuracy. This pattern strongly suggests that the observed gains can be attributed to the application of treatment. In sum, all participants were judged to demonstrate sufficiently stable baselines to serve as the basis for an evaluation of the effects of treatment.

For convenience, the single-subject graphs have been grouped into three sets of 3–4 participants who demonstrated broadly similar patterns of response to treatment. Within each group, participants are ordered by increasing length of the baseline phase. **Figure 2** shows four participants for whom visual inspection offered strong evidence of a response to at least one type of treatment. Visual inspection of data from 10;2-year-old Aiden shows a large change in level between the baseline phase and all subsequent phases of treatment, with minimal overlap between values observed in the baseline phase versus any subsequent phase. This change occurred immediately after the initiation of treatment. Aiden’s progress in the first phase, which featured traditional articulatory treatment, yielded a large *ES_Phase1_* of 7.28. His performance declined to some extent in the second phase, which featured biofeedback treatment, yielding an *ES_Phase2_* of -2.44 from midpoint to maintenance. However, his performance still remained substantially above baseline levels, with a final *ES_All_* of 5.22.

Ella, age 13;8, also showed a sizable change in level between the baseline phase and all subsequent phases of the study. This change was evident in post-treatment word probes within the first three sessions of treatment; from the fourth session on, there was virtually no overlap of data points with the baseline phase. The first phase, which featured biofeedback treatment, yielded an *ES_Phase1_* of 4.34. Ella’s gains remained mostly stable through the second phase of treatment and the maintenance period, yielding an *ES_All_* of 3.4.

Participants Landon, age 9;6, and Mason, age 12;10, exhibited similar trajectories of progress, although Mason showed higher overall accuracy throughout the study. Both boys began in the biofeedback treatment condition and began to show gains within the first three sessions of treatment; they sustained their gains during midpoint probes and made additional improvements in the second phase of treatment. They likewise showed no overlap between the baseline phase and data points in the midpoint phase, second phase of treatment, or maintenance phase. In Mason’s case, there also was very little overlap between the baseline phase and the first phase of treatment. For both boys, a larger effect size was calculated for the first phase of treatment, in which biofeedback was provided, although the smaller gains in the second phase could be attributed to a ceiling effect in Mason’s case. Large overall effect sizes of 7.79 and 20.45 were observed for Landon and Mason, respectively.

**Figure 3** shows three participants for whom visual inspection offered moderate evidence of a response to at least one type of treatment. Erica, age 9;3, showed no visual evidence of improvement in the first phase, which featured traditional treatment. (An *ES_Phase1_* of 3.12 was computed for her, but given that the raw difference in 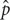
_correct_ was only 3.02, this was judged to be an artifact of low variance during the baseline phase.) During the second phase of treatment, Erica showed a change in trend from a stable near-zero level of accuracy to a small but consistent increase. This rising trend continued into the maintenance phase, suggesting that ongoing generalization gains might be anticipated. Her final effect size (*ES_All_*) of 7.01 reflected robust overall gains, although her scores in the maintenance phase remained well below ceiling-level accuracy.

For participant Jason, age 15;10, no meaningful change was evident during the first phase of treatment, which featured biofeedback. He also showed no change throughout most of the second treatment phase, but in the final few sessions, his perceptually rated accuracy took on a distinct upward slope. These gains tended to affect post-test but not pre-test probes, and accuracy was not sustained throughout the maintenance period. This suggests more short-term learning that was not yet robustly transferring to other contexts. Accordingly, the effect sizes calculated using midpoint and maintenance probe data suggested a weaker effect of treatment than visual inspection of within-treatment probe data would suggest (*ES_Phase1_* 0.07; *ES_Phase2_* 1.19; *ES_All_* 1.41).

Participant Gregory, age 9;10, showed a small increase in the perceptually rated accuracy of rhotics produced during Phase 1, which featured biofeedback treatment, followed by a slightly larger increase during Phase 2. Ongoing overlap in data points between baseline and treatment phases prevents us from attaching a strong interpretation to these changes, and gains were minimally sustained into the midpoint and maintenance probe intervals. All three effect sizes computed for Gregory fell short of the threshold to be considered clinically significant (*ES_Phase1_* 0.03; *ES_Phase2_* 0.66; *ES_All_* 0.7). On the other hand, Gregory’s accuracy during both phases of treatment was far more variable than at baseline. This suggested that he was engaging some degree of exploration of new strategies for rhotic production, although he had not yet stabilized a production pattern that would reliably yield a perceptually accurate rhotic sound.

Finally, **Figure 4** depicts the four participants for whom visual inspection of generalization probe data yielded no significant evidence of a response to either type of treatment. Three of these participants (Bryce, Holly, and Harper) began treatment in the traditional treatment condition, while one participant (Cooper) received treatment in the opposite order. For all of these participants, visual inspection of perceptual rating data yields a consistent picture of minimal change across all phases of the study. Effect sizes are generally in accordance with visual inspection; an exception is Cooper’s *ES_Phase1_* of 2.0, but this value is inflated by minimal variance during the baseline phase. Note also that most participants in this group had near-zero perceptual accuracy ratings at baseline, contrasting with the more intermediate accuracy ratings given to participants in the other groups; we return to this topic in the Section “Discussion.”

### Across-Subjects Comparisons: Effect Sizes

**FIGURE 5:**
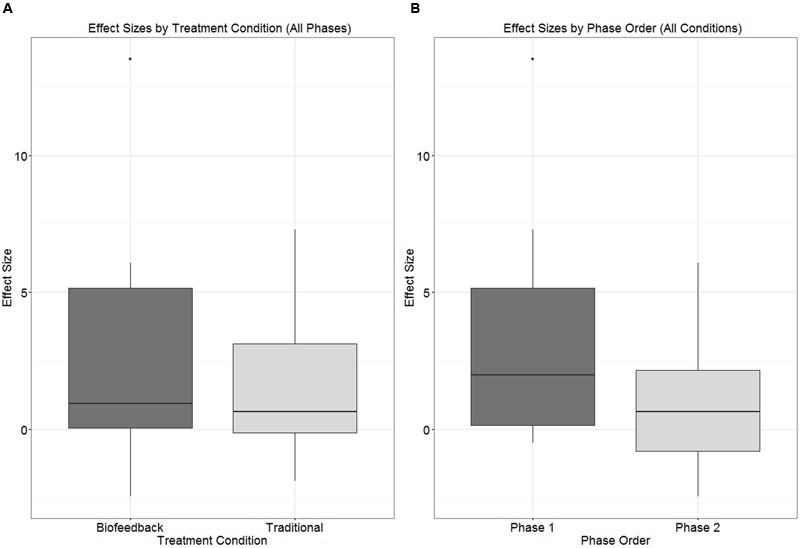
**Boxplots depicting the distribution of effect sizes observed in connection with (A)** biofeedback versus traditional treatment condition, independent of phase; **(B)** Phase 1 versus Phase 2 of treatment, independent of treatment condition.

**FIGURE 6:**
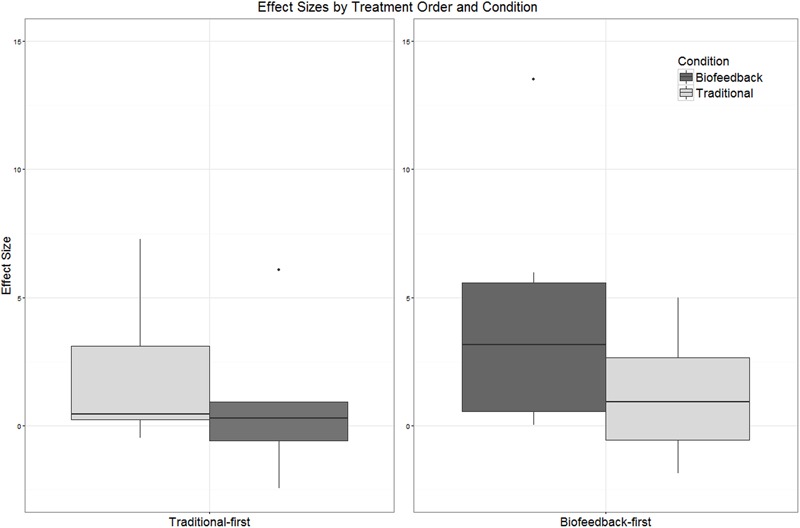
**Boxplots depicting the distribution of effect sizes observed in connection with biofeedback versus traditional treatment.** Participants have been partitioned into two groups reflecting the order in which treatment was delivered (traditional-first versus biofeedback-first).

Although the primary focus of this single-subject experimental study is on within-subject changes, the counterbalanced design makes it possible to explore between-subjects effects as well. The boxplots in **Figures 5** and **6** depict the distribution of effect sizes observed when the data are partitioned in different ways. In **Figure 5A**, effect sizes associated with biofeedback treatment (*ES_BF_*) are compared against effect sizes from traditional treatment (*ES_Trad_*), independent of the order in which the two types of treatment were delivered. **Figure 5A** shows that the effect size distributions observed in biofeedback versus traditional phases of treatment are extensively overlapped and have very similar median values, although the interquartile range extends to somewhat higher effect sizes for biofeedback than traditional treatment. **Figure 5B** examines a possible order effect, comparing the distribution of values of *ES_Phase1_* versus *ES_Phase2_*, independent of the type of treatment delivered in each of those phases. This figure shows that effect sizes tended to be larger in the first phase of treatment than in the second phase, although again there is considerable overlap between the interquartile ranges for the two distributions.

Strong differences between the treatment conditions become apparent only when we distinguish among participants based on the order in which the two treatment types were provided. In **Figure 6**, participants are partitioned into those who received traditional treatment first versus those who received biofeedback treatment first, and the distribution of effect sizes observed in each treatment condition is plotted. Effect sizes observed in connection with biofeedback treatment were substantially larger when biofeedback was provided in Phase 1 than in Phase 2. For the traditional treatment condition, the median effect size was slightly higher when traditional treatment was provided in Phase 2 versus Phase 1, but the interquartile ranges between the two cases overlap almost completely. Due to the small number of data points, hypothesis tests were not conducted on these comparisons of effect sizes across treatment conditions. Instead, the logistic mixed model reported in the next section will examine these effects and their interactions in greater detail.

### Across-Subjects Comparisons: Logistic Mixed-Effects Model

**FIGURE 7:**
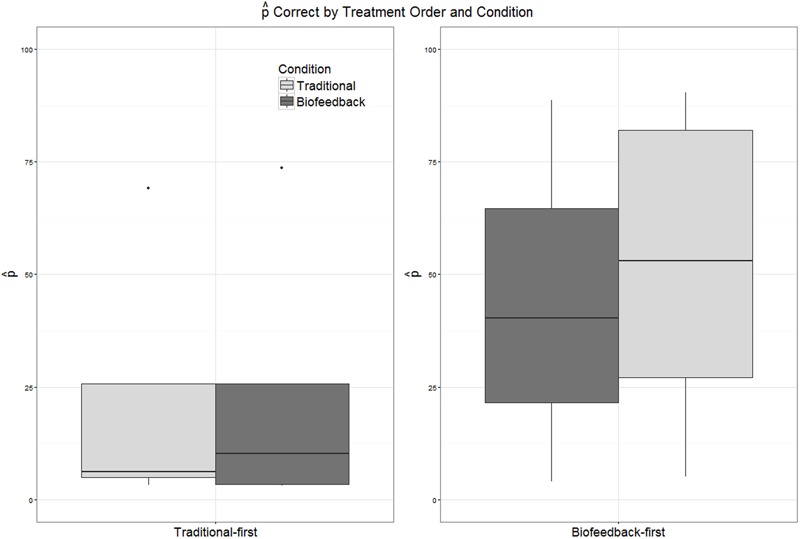
**Boxplots depicting the distribution of values of 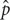
_correct_ in pre- and post-treatment probes administered during biofeedback versus traditional treatment.** Participants have been partitioned into two groups reflecting the order in which treatment was delivered (traditional-first versus biofeedback-first).

As described in the “Analyses” section, a logistic mixed-effects model was fitted over all data points from both pre- and post-treatment probes elicited during the two phases of treatment. Candidate model structures were compared using likelihood ratio tests, and predictors that did not make a significant contribution were dropped. The final reduced model included fixed effects of accuracy in the baseline phase, treatment condition, and order of treatment delivery, as well as the first-order interaction between treatment condition and order of treatment delivery. A by-subject random slope for the effect of treatment condition was also included. Unsurprisingly, mean accuracy in the pre-treatment baseline phase, as assessed by blinded listeners, was a significant predictor of accuracy in probes administered over the course of treatment (β = 2.11, *SE* = 0.14, *p* < 0.001). Treatment condition (traditional versus biofeedback) was not a significant predictor of rhotic production accuracy (β = -0.11, *SE* = 0.22, *p* = 0.63), nor was order of treatment delivery (β = -0.04, *SE* = 0.28, *p* = 0.88). However, the interaction between treatment condition and treatment order was significant (β = 0.67, *SE* = 0.30, *p* = 0.03). This interaction can be visualized in **Figure 7**, which plots accuracy in pre- and post-treatment probes during both biofeedback and traditional treatment conditions; they are pooled across subjects but partitioned by the order of treatment application (biofeedback-first or traditional-first). Raw counts of ratings have been converted to 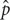
_correct_ for equivalence with other figures in the paper. **Figure 7** shows that the overall highest accuracy was observed during a phase of traditional treatment that came after a phase of biofeedback treatment. The second-highest accuracy was observed in an initial phase of biofeedback treatment. Thus, in this data set, biofeedback appeared to have greater efficacy than traditional articulatory methods when provided in the first phase of treatment, and traditional methods appeared to have greater efficacy than biofeedback when provided in the second phase of treatment. Lastly, **Figure 7** shows that accuracy scores tended to be higher in the biofeedback-first order than the traditional-first order, although this effect was not significant.

## Discussion

### Overall Response to Treatment

This study aimed to compare the relative contribution of visual-acoustic biofeedback versus traditional treatment in a two-phase, counterbalanced intervention package for children with residual errors affecting rhotic production. However, we begin our discussion by addressing a more basic question: did participants show evidence of a response to either phase of treatment, and/or to the combined treatment package? Based on a combination of visual inspection and calculation of effect sizes, we conclude that seven out of eleven participants showed evidence of a meaningful response to at least one type of treatment, while four participants showed no evidence of a response to either type of treatment. Of the seven participants who responded to treatment, four were judged to show strong visual evidence of an intervention effect, with moderate to large overall effect sizes. Three were judged to show moderate visual evidence of an effect, with small to moderate effect sizes. Given that participants had previously received a median duration of 2 years of treatment targeting rhotics without success, and the present treatment was only 10 weeks in duration, it is reasonable to describe the combined treatment package as an effective form of intervention for residual rhotic errors.

The present results are in keeping with many previous studies of biofeedback intervention, where it is typical to find a diverse range of individual responses to treatment, including non-responders (e.g., McAllister Byun et al., 2014b; Preston et al., 2014). This high degree of variability suggests that some individuals may be better suited to benefit from biofeedback intervention than others. However, previous research has not succeeded in identifying individual characteristics that reliably indicate which candidates are most likely to demonstrate a successful response to biofeedback treatment. Unfortunately, the present study is no exception: there were no significant relationships between overall effect size and demographic variables including age (ρ = 0.06, *p* = 0.87), duration of previous treatment targeting rhotics (ρ = -0.41, *p* = 0.27), or accuracy in the baseline phase (ρ = 0.45, *p* = 0.16). The non-significance of this last correlation was somewhat surprising; visual inspection of **Figures 2**–**4** suggests that the participants who exhibited the strongest response to treatment were judged by naïve listeners to produce rhotics with moderate accuracy in the baseline period, while participants who showed no response to treatment were mostly judged to exhibit near-zero accuracy across the baseline phase. It is possible that a significant correlation between baseline accuracy and treatment response would emerge in a larger sample of participants. In general, it is clear that larger-scale research, including correlational studies that aggregate data over multiple smaller treatment studies, will be necessary to identify factors that can predict the likelihood that a given individual with residual rhotic errors will respond to treatment.

### Differential Response to Traditional versus Biofeedback Treatment

Visual inspection of individual participants’ results yielded no conclusive evidence of a difference in efficacy between biofeedback and traditional conditions. There were instances of participants who responded to traditional but not biofeedback treatment (Aiden, Jason), participants who responded to biofeedback but not traditional treatment (Erica, Ella), and participants who responded to both or to neither treatment. Contrary to hypothesis, the effect sizes observed in connection with biofeedback treatment phases did not differ significantly from the effect sizes observed for traditional treatment phases (**Figure 5A**), and the mixed logistic model yielded no main effect of traditional versus biofeedback treatment condition. On the other hand, the mixed logistic model did show a significant interaction between treatment condition and the order in which treatments were administered. Initial phases of biofeedback treatment were associated with a high level of performance, and traditional treatment phases that occurred following a biofeedback treatment phase were associated with the highest accuracy of all. The lowest level of accuracy was observed in initial traditional treatment phases, with only a small increase in accuracy in the biofeedback treatment phases that followed.

One possibility is that this interaction was a chance occurrence. Given the small number of participants, it could be that the random assignment of participants to treatment orders happened to allocate a disproportionate number of highly treatment-resistant participants to the traditional-first condition. On the other hand, the group of individuals randomly allocated to the biofeedback-first condition did not differ significantly from the traditional-first group with respect to baseline accuracy (*t* = -1.3, *df* = 9.0, *p* = 0.23), age (*t* = -1.49, df = 8.0, *p* = 0.18), or duration of previous treatment (*t* = 0.79, *df* = 5.7. *p* = 0.46). In addition, the interaction was significant in a model that accounted for differences in baseline accuracy. Furthermore, the suggestion that biofeedback may have an advantage over traditional treatment only in early stages of treatment is very much in keeping with one theoretical model of how biofeedback could influence motor learning. As discussed in the introduction, biofeedback provides a detailed form of KP feedback. As such, it is hypothesized to be most advantageous in the earliest stages of learning, when the target motor routine is still being established. For motor skills to generalize to other contexts, though, the learner must be able to evaluate his/her own accuracy without depending on detailed KP feedback. It has therefore been argued that KP feedback becomes ineffective or even detrimental in later stages of motor learning (Hodges and Franks, 2001). It follows that any advantage for biofeedback over traditional treatment could be expected to manifest in early stages of treatment, potentially disappearing at later stages.

It is noteworthy that participants in the biofeedback-first condition were able to achieve a high level of accuracy in rhotic production even though they received no cues regarding articulator placement. As discussed in McAllister Byun et al. (2016b), it is commonly assumed that explicit placement cues are essential for achieving accurate production. These results support McAllister Byun et al. (2016b) and other recent research (e.g., Rvachew and Brosseau-Lapré, 2012) in finding that correct production can in some cases be achieved through intervention that focuses exclusively on the acoustic or auditory properties of a target sound. These results are also in keeping with theoretical models that maintain that speech targets are primarily acoustic rather than articulatory in nature (e.g., Guenther et al., 1998; Perkell, 2012). Note that we do not mean to suggest that explicit articulatory cues are detrimental or should be avoided. Current evidence suggests that clinicians can choose whether or not to incorporate such cues based on their own preferences and those of the client.

## Future Directions

We undertook this research with the hypothesis that comparison of biofeedback and traditional treatment phases would reveal an advantage for the biofeedback treatment condition. Although this hypothesis was not supported, we come away from the study with a refined hypothesis that biofeedback should show an advantage over traditional methods in early stages of treatment. A logical follow-up to the present experiment would be a similar study in which participants are randomly assigned to receive a phase of biofeedback treatment followed by traditional treatment, versus an equal duration featuring exclusively traditional treatment. If such a follow-up were to support the present study in finding a facilitative effect of an initial period of biofeedback treatment, subsequent manipulations should aim to determine the optimal duration of biofeedback treatment to provide prior to the transition to traditional methods. It should be kept in mind that the present study incorporated adaptive changes in treatment difficulty that were specifically designed to avoid excessive dependence on external feedback. The first adjustment in difficulty, which took effect as soon as a participant was judged to produce perceptually accurate rhotics in at least 8/10 consecutive trials, took the form of a reduction from 100 to 50% frequency of external feedback. It is possible that an earlier reduction in feedback frequency would have been more facilitative, or that the total duration of biofeedback practice should be limited in order to avoid excessive dependence.

One important modification to incorporate into future research is an increase in dose frequency, i.e. the number of trials elicited per session. The present dose frequency of 60 trials per session was determined based on previous biofeedback research (e.g., McAllister Byun and Hitchcock, 2012; McAllister Byun et al., 2014b). However, participants in those studies were as young as 6;0, while all participants in the present study were 9;0 or older. Older participants are capable of producing a larger number of trials per session. Because larger numbers of practice trials have been observed to yield larger treatment effects (e.g., Edeal and Gildersleeve-Neumann, 2011), a higher dose of treatment might reduce the number of non-responders and allow a clearer picture of response to treatment to emerge.

## Conclusion

Biofeedback technologies to enhance speech intervention have received considerable attention in the literature, and numerous studies have suggested that biofeedback may succeed in eliminating residual speech errors in individuals who have not responded to other forms of treatment. However, there is a lack of controlled experimental evidence comparing the efficacy of biofeedback to non-biofeedback interventions. The present study used a single-subject experimental design with a crossover component to document the relative efficacy of visual-acoustic biofeedback versus traditional articulatory treatment for residual rhotic errors. Out of 11 participants, seven were judged to exhibit evidence of a response to at least one type of treatment. Contrary to expectation, neither individual data nor a mixed-effects logistic regression revealed a reliable advantage for biofeedback over traditional phases of treatment. However, there was a significant interaction between treatment condition and treatment order: biofeedback appeared to be more effective than traditional articulatory methods in the first phase of treatment, and traditional treatment appeared to have greater efficacy than biofeedback in the second phase of the study. This is consistent with existing findings in the literature exploring principles of motor learning (e.g., Maas et al., 2008). Specifically, as a detailed form of KP feedback, biofeedback is predicted to be advantageous in early stages of treatment, but this advantage may be attenuated or eliminated as the goal of treatment shifts from acquisition to generalization of an accurate motor plan. Further research should test this refined hypothesis while also exploring what duration of biofeedback treatment might yield optimal treatment effects.

## Author Contributions

Designed the study: TMB. Collected and analyzed data: TMB and HC. Wrote the manuscript: TMB and HC.

## Conflict of Interest Statement

The authors declare that the research was conducted in the absence of any commercial or financial relationships that could be construed as a potential conflict of interest.

## Footnotes

1 Because the present study focuses on visual-acoustic rather than articulatory biofeedback intervention for rhotic errors, we will not go into extensive detail regarding the articulatory characteristics of rhotics; readers are referred to Adler-Bock et al. (2007), Klein et al. (2013), or Boyce (2015) for in-depth discussion.

2 In addition to conveying information about sonorant identity, formant frequencies also reflect the height and sex of the speaker (e.g., Lee et al., 1999). Model productions used as targets in biofeedback should thus be drawn from a speaker whose vocal tract is comparable in size to that of a particular learner. In the current study, participants were prompted to sustain the vowels /i/, /ɑ/, and /u/ so that the clinician could find the best matches to the formant patterns of those vowels in a library of templates collected from children of different ages. The /ɹ/ template from the model speaker whose non-rhotic vowels were the best match for a particular participant was used as the treatment target for that participant.

3 If both members of a pair of consonants that differed only in voicing (e.g. /s, z/) were affected in the same way, this was counted as a single error.

4 Although we are reporting percentages, we use the label _correct_ as a reminder that these values are distinct from, but correlated with, typical “percent correct” measures.

5 Due to a server error, specific demographic data could not be retrieved for 76 participants. However, we do know that these participants did not report any characteristics considered grounds for exclusion, or they would not have been permitted by the software to proceed to the rating task.

6 The mean number of probe words on which _correct_ scores are based was 14.98 (*SD* = 0.25) for pre- and post-treatment probes and 30 (*SD* = 0.0) for baseline and maintenance probes. The number of ratings collected in connection with a given probe session, i.e., the denominator in _correct_, was roughly nine times the number of items in that probe and often was larger.

## References

[B1] Adler-Bock M. Bernhardt B. M. Gick B. Bacsfalvi P. (2007). The use of ultrasound in remediation of North American English /r/ in 2 adolescents. *Am. J. Speech Lang. Pathol.* 16 128–139. 10.1044/1058-0360(2007/017) 17456891

[B2] Bacsfalvi P. (2010). Attaining the lingual components of /r/ with ultrasound for three adolescents with cochlear implants. *Rev. Can. Orthoph. Audiol.* 34:206.

[B3] Beeson P. M. Robey R. R. (2006). Evaluating single-subject treatment research: Lessons learned from the aphasia literature. *Neuropsychol. Rev.* 16 161–169. 10.1007/s11065-006-9013-7 17151940 PMC2366174

[B4] Bislick L. P. Weir P. C. Spencer K. Kendall D. Yorkston K. M. (2012). Do principles of motor learning enhance retention and transfer of speech skills? A systematic review. *Aphasiology* 26 709–728. 10.1080/02687038.2012.676888

[B5] Boyce S. Espy-Wilson C. (1997). Coarticulatory stability in American English /r/. *J. Acoustic. Soc. Am.* 101 3741–3753. 10.1121/1.418333 9193061

[B6] Boyce S. E. (2015). The articulatory phonetics of /r/ for residual speech errors. *Semin. Speech Lang.* 36 257–270. 10.1055/s-0035-1562909 26458201 PMC4915106

[B7] Brossart D. F. Parker R. I. Olson E. A. Mahadevan L. (2006). The relationship between visual analysis and five statistical analyses in a simple AB single-case research design. *Behav. Modif.* 30 531–563. 10.1177/0145445503261167 16894229

[B8] Crump M. J. McDonnell J. V. Gureckis T. M. (2013). Evaluating Amazon’s mechanical turk as a tool for experimental behavioral research. *PLoS ONE* 8:e57410 10.1371/journal.pone.0057410 PMC3596391 23516406

[B9] Davis S. M. Drichta C. E. (1980). Biofeedback theory and application in allied health. *Biofeedback Self-Regul.* 5 159–174. 10.1007/BF00998593 6994823

[B10] Delattre P. Freeman D. C. (1968). A dialect study of American r’s by x-ray motion picture. *Linguistics* 44 29–68.

[B11] Edeal D. M. Gildersleeve-Neumann C. E. (2011). The importance of production frequency in therapy for childhood apraxia of speech. *Am. J. Speech Lang. Pathol.* 20 95–110. 10.1044/1058-0360(2011/09-0005) 21330650

[B12] Flipsen P. Jr. Shriberg L. D. Weismer G. Karlsson H. B. McSweeny J. L. (2001). Acoustic phenotypes for speech-genetics studies: reference data for residual /ɝ/ distortions. *Clin. Ling. Phon.* 15 603–630. 10.1080/02699200110069410 12469448

[B13] Gibbon F. Paterson L. (2006). A survey of speech and language therapists’ views on electropalatography therapy outcomes in Scotland. *Child Lang. Teach. Ther.* 22 275–292. 10.1191/0265659006ct308xx

[B14] Gibbon F. Stewart F. Hardcastle W. J. Crampin L. (1999). Widening access to electropalatography for children with persistent sound system disorders. *Am. J. Speech Lang. Pathol.* 8 319–334. 10.1044/1058-0360.0804.319

[B15] Goldman R. Fristoe M. (2000). *Goldman-Fristoe Test of Articulation* 2nd Edn. Circle Pines, MN: AGS.

[B16] Guadagnoli M. A. Lee T. D. (2004). Challenge point: a framework for conceptualizing the effects of various practice conditions in motor learning. *J. Mot. Behav.* 36 212–224. 10.3200/JMBR.36.2.212-224 15130871

[B17] Guenther F. H. Hampson M. Johnson D. (1998). A theoretical investigation of reference frames for the planning of speech movements. *Psychol. Rev.* 105 611–633. 10.1037/0033-295X.105.4.611-633 9830375

[B18] Hagiwara R. (1995). Acoustic realizations of American /r/ as produced by women and men. *UCLA Work. Papers Phon.* 90 1–187.

[B19] Hitchcock E. R. Harel D. McAllister Byun T. (2015). Social, emotional, and academic impact of residual speech errors in school-age children: a survey study. *Semin. Speech Lang.* 36 283–294. 10.1055/s-0035-1562911 26458203 PMC5708870

[B20] Hitchcock E. R. McAllister Byun T. (2014). Enhancing generalization in biofeedback intervention using the challenge point framework: a case study. *Clin. (Ling.) Phon.* 29 59–75. 10.3109/02699206.2014.956232 PMC4276132 25216375

[B21] Hodges N. J. Franks I. M. (2001). Learning a coordination skill: interactive effects of instruction and feedback. *Res. Q. Exer. Sport* 72 132–142. 10.1080/02701367.2001.10608943 11393876

[B22] Ipeirotis P. G. Provost F. Sheng V. S. Wang J. (2014). Repeated labeling using multiple noisy labelers. *Data Min. Knowl. Discov.* 28 402–441. 10.1007/s10618-013-0306-1

[B23] Kaderavek J. N. Justice L. M. (2010). Fidelity: an essential component of evidence-based practice in speech-language pathology. *Am. J. Speech Lang. Pathol.* 19 369–379. 10.1044/1058-0360(2010/09-0097) 20601624

[B24] Katz W. Mcneil M. And Garst D. (2010). Treating apraxia of speech (AOS) with EMA-supplied visual augmented feedback. *Aphasiology* 24 826–837. 10.1080/02687030903518176

[B25] Klein H. B. McAllister Byun T. Davidson L. Grigos M. I. (2013). A multidimensional investigation of children’s /r/ productions: perceptual, ultrasound, and acoustic measures. *Am. J. Speech Lang. Pathol.* 22 540–553. 10.1044/1058-0360(2013/12-0137) 23813195 PMC4266408

[B26] Kratochwill T. R. Hitchcock J. Horner R. H. Levin J. R. Odom S. L. Rindskopf D. M. (2010). *Single-Case Designs Technical Documentation.* Available at: http://ies.ed.gov/ncee/wwc/Document/229

[B27] Kratochwill T. R. Levin J. R. (2014). *Single-Case Intervention Research: Methodological and Statistical Advances*. Washington, DC: American Psychological Association.

[B28] Kratochwill T. R. Levin J. R. Horner R. H. Swoboda C. M. (2014). “Visual analysis of single-case intervention research: conceptual and methodological issues,” in *Single-Case Intervention Research: Methodological and Data-Analysis Advances* eds Kratochwill T. R. Levin J. R. (Washington, DC: American Psychological Association).

[B29] Lee S. Potamianos A. Narayanan S. (1999). Acoustics of children’s speech: developmental changes of temporal and spectral parameters. *J. Acoust. Soc. Am.* 105 1455–1468. 10.1121/1.426686 10089598

[B30] Lockenvitz S. Kuecker K. Ball M. J. (2015). Evidence for the distinction between “consonantal-/r/” and “vocalic-/r/” in American English. *Clin. Ling. Phon.* 29 613–622. 10.3109/02699206.2015.1047962 26172586

[B31] Maas E. Farinella K. A. (2012). Random versus blocked practice in treatment for childhood apraxia of speech. *J. Speech Lang. Hear. Res.* 55 561–578. 10.1044/1092-4388(2011/11-0120) 22207698

[B32] Maas E. Robin D. A. Austermann Hula S. N. Freedman S. E. Wulf G. Ballard K. J. (2008). Principles of motor learning in treatment of motor speech disorders. *Am. J. Speech Lang. Pathol.* 17 277–298. 10.1044/1058-0360(2008/025) 18663111

[B33] Magloughlin L. (2016). Accounting for variability in North American English /ɹ/: Evidence from children’s articulation. *J. Phon.* 54 51–67. 10.1016/j.wocn.2015.07.007

[B34] Martin N. A. Brownell R. (2005). *Test of Auditory Processing Skills* 3rd Edn. Novato, CA: Academy Therapy Publications.

[B35] McAllister Byun T. Halpin P. F. Szeredi D. (2015). Online crowdsourcing for efficient rating of speech: a validation study. *J. Commun. Disord.* 53 70–83. 10.1016/j.jcomdis.2014.11.003 25578293 PMC4346507

[B36] McAllister Byun T. Harel D. Halpin P. F. Szeredi D. (2016a). Deriving gradient measures of child speech from crowdsourced ratings. *Forthcoming J. Commun. Disord.* 10.1016/j.jcomdis.2016.07.001 [Epub ahead of print]. PMC5553126 27481555

[B37] McAllister Byun T. Hitchcock E. R. (2012). Investigating the use of traditional and spectral biofeedback approaches to intervention for /r/ misarticulation. *Am. J. Speech Lang. Pathol.* 21 207–221. 10.1044/1058-0360(2012/11-0083) 22442281

[B38] McAllister Byun T. Hitchcock E. R. Ortiz J. (2014a). Challenge-R: computerized challenge point treatment for /r/ misarticulation. *Paper Presented at ASHA 2014* Orlando, FL.

[B39] McAllister Byun T. Hitchcock E. R. Swartz M. T. (2014b). Retroflex versus bunched in treatment for /r/ misarticulation: evidence from ultrasound biofeedback intervention. *J. Speech Lang. Hear. Res.* 57 2116–2130. 10.1044/2014_JSLHR-S-14-0034 25088034 PMC4294189

[B40] McAllister Byun T. Swartz M. T. Halpin P. F. Szeredi D. Maas E. (2016b). Direction of attentional focus in biofeedback treatment for /r/ misarticulation. *Int. J. Lang. Commun. Disord.* 51 384–401. 10.1111/1460-6984.12215 26947142 PMC4931951

[B41] McCormack J. McLeod S. McAllister L. Harrison L. J. (2009). A systematic review of the association between childhood speech impairment and participation across the lifespan. *Int. J. Speech Lang. Pathol.* 11 155–170. 10.1080/17549500802676859

[B42] McGowan R. S. Nittrouer S. Manning C. J. (2004). Development of [r] in young, midwestern. *Am. Child. J. Acoustic. Soc. Am.* 115 871–884. 10.1121/1.1642624 PMC3987658 15000198

[B43] Paolacci G. Chandler J. Ipeirotis P. (2010). Running experiments on Amazon Mechanical Turk. *Judg. Decis. Mak.* 5 411–419.

[B44] Perkell J. S. (2012). Movement goals and feedback and feedforward control mechanisms in speech production. *J. Neuroling.* 25 382–407. 10.1016/j.jneuroling.2010.02.011 PMC3361736 22661828

[B45] Preston J. L. Brick N. Landi N. (2013). Ultrasound biofeedback treatment for persisting childhood apraxia of speech. *Am. J. Speech Lang. Pathol.* 22 627–643. 10.1044/1058-0360(2013/12-0139) 23813207

[B46] Preston J. L. Leece M. C. Maas E. (2016). Motor-based treatment with and without ultrasound feedback for residual speech-sound errors. *Int. J. Lang. Commun. Disord.* 10.1111/1460-6984.12259 [Epub ahead of print]. PMC5156595 27296780

[B47] Preston J. L. Mccabe P. Rivera-Campos A. Whittle J. L. Landry E. Maas E. (2014). Ultrasound visual feedback treatment and practice variability for residual speech sound errors. *J. Speech Lang. Hear. Res.* 57 2102–2115. 10.1044/2014_JSLHR-S-14-0031 25087938 PMC4272648

[B48] Rindskopf D. Ferron J. (2014). “Using multilevel models to analyze single-case design data,” in *Single-Case Intervention Research: Methodological and Statistical Advances* eds Kratochwill T. R. Levin J. R. (Washington, DC: American Psychological Association) 221–246.

[B49] Robey R. R. (2004). A five-phase model for clinical-outcome research. *J. Commun. Disord.* 37 401–411. 10.1016/j.jcomdis.2004.04.003 15231420

[B50] Ruscello D. M. (1995). Visual feedback in treatment of residual phonological disorders. *J. Commun. Disord.* 28 279–302. 10.1016/0021-9924(95)00058-Xx2 8576411

[B51] Rvachew S. Brosseau-Lapré F. (2012). *Developmental Phonological Disorders: Foundations of Clinical Practice*. San Diego, CA: Plural Publishing.

[B52] Shipley K. McAfee J. (2008). *Assessment in Speech-Language Pathology: A Resource Manual*. Boston, MA: Cengage Learning.

[B53] Shriberg L. D. (2010). “Childhood speech sound disorders: from post-behaviorism to the post-genomic era,” in *Speech Sound Disorders in Children* eds Paul R. Flipsen P. (San Diego, CA: Plural Publishing) 1–34.

[B54] Shriberg L. D. Flipsen P. J. Karlsson H. B. McSweeny J. L. (2001). Acoustic phenotypes for speech-genetics studies: an acoustic marker for residual /r/ distortions. *Clin. Ling. Phon.* 15 631–650. 10.1080/02699200110069429 12469448

[B55] Shriberg L. D. Gruber F. A. Kwiatkowski J. (1994). Developmental phonological disorders III: long-term speech-sound normalization. *J. Speech Lang. Hear. Res.* 37 1151–1177. 10.1044/jshr.3705.1127 7823558

[B56] Shriberg L. D. Kwiatkowski J. Hoffmann K. (1984). A procedure for phonetic transcription by consensus. *J. Speech Lang. Hear. Res.* 27 456–465. 10.1044/jshr.2703.456 6482415

[B57] Shuster L. I. Ruscello D. M. Smith K. D. (1992). Evoking [r] using visual feedback. *Am. J. Speech Lang. Pathol.* 1 29–34. 10.1044/1058-0360.0103.29

[B58] Shuster L. I. Ruscello D. M. Toth A. R. (1995). The use of visual feedback to elicit correct /r/. *Am. J. Speech Lang. Pathol.* 4 37–44. 10.1044/1058-0360.0402.37

[B59] Smit A. B. Hand L. Freilinger J. J. Bernthal J. E. Bird A. (1990). The Iowa articulation norms project and its Nebraska replication. *J. Speech Hear. Disord.* 55 779–798. 10.1044/jshd.5504.779 2232757

[B60] Sprouse J. (2011). A validation of Amazon Mechanical Turk for the collection of acceptability judgments in linguistic theory. *Behav. Res. Methods* 43 155–167. 10.3758/s13428-010-0039-7 21287108 PMC3048456

[B61] Van Riper C. Erickson R. L. (1996). *Speech Correction: An Introduction to Speech Pathology and Audiology* 9th Edn. Englewood Cliffs, NJ: Prentice-Hall.

[B62] Volin R. A. (1998). A relationship between stimulability and the efficacy of visual biofeedback in the training of a respiratory control task. *Am. J. Speech Lang. Pathol.* 7 81–90. 10.1044/1058-0360.0701.81

