# Supplementary material for: Differential Effects of Visual-Acoustic Biofeedback Intervention for Residual Speech Errors
Source: Front Hum Neurosci. 2016 Nov 11;10:567. doi: 10.3389/fnhum.2016.00567 (PMC5104733; doi:10.3389/fnhum.2016.00567)
Supplement: Supplementary file 1 [file Data_Sheet_1.DOCX]

**Appendix A. Rhotic Probe Words**

| **Word** | **Consonantal or Vocalic** | **Included in which probes** |
| --- | --- | --- |
| rose | Consonantal | Baseline/maintenance, within-treatment (pre and post) |
| rude | Consonantal | Baseline/maintenance, within-treatment (pre and post) |
| raw | Consonantal | Baseline/maintenance, within-treatment (pre and post) |
| rob | Consonantal | Baseline/maintenance, within-treatment (pre and post) |
| rug | Consonantal | Baseline/maintenance, within-treatment (pre and post) |
| race | Consonantal | Baseline/maintenance, within-treatment (pre and post) |
| red | Consonantal | Baseline/maintenance, within-treatment (pre and post) |
| ride | Consonantal | Baseline/maintenance, within-treatment (pre and post) |
| reach | Consonantal | Baseline/maintenance, within-treatment (pre and post) |
| raft | Consonantal | Baseline/maintenance, within-treatment (pre and post) |
| scarf | Vocalic | Baseline/maintenance, within-treatment (pre and post) |
| dark | Vocalic | Baseline/maintenance, within-treatment (pre and post) |
| farm | Vocalic | Baseline/maintenance, within-treatment (pre and post) |
| chair | Vocalic | Baseline/maintenance, within-treatment (pre and post) |
| stare | Vocalic | Baseline/maintenance, within-treatment (pre and post) |
| weird | Vocalic | Baseline/maintenance, within-treatment (pre and post) |
| clear | Vocalic | Baseline/maintenance, within-treatment (pre and post) |
| beard | Vocalic | Baseline/maintenance, within-treatment (pre and post) |
| fork | Vocalic | Baseline/maintenance, within-treatment (pre and post) |
| sword | Vocalic | Baseline/maintenance, within-treatment (pre and post) |
| turn | Vocalic | Baseline/maintenance, within-treatment (pre and post) |
| nurse | Vocalic | Baseline/maintenance, within-treatment (pre and post) |
| worm | Vocalic | Baseline/maintenance, within-treatment (pre and post) |
| ladder | Vocalic | Baseline/maintenance, within-treatment (pre and post) |
| hammer | Vocalic | Baseline/maintenance, within-treatment (pre and post) |
| rock | Consonantal | Baseline/maintenance only |
| wrong | Consonantal | Baseline/maintenance only |
| robe | Consonantal | Baseline/maintenance only |
| run | Consonantal | Baseline/maintenance only |
| rules | Consonantal | Baseline/maintenance only |
| read | Consonantal | Baseline/maintenance only |
| ring | Consonantal | Baseline/maintenance only |
| rake | Consonantal | Baseline/maintenance only |
| wrap | Consonantal | Baseline/maintenance only |
| rip | Consonantal | Baseline/maintenance only |
| barn | Vocalic | Baseline/maintenance only |
| star | Vocalic | Baseline/maintenance only |
| board | Vocalic | Baseline/maintenance only |
| door | Vocalic | Baseline/maintenance only |
| floor | Vocalic | Baseline/maintenance only |
| scare | Vocalic | Baseline/maintenance only |
| share | Vocalic | Baseline/maintenance only |
| tear | Vocalic | Baseline/maintenance only |
| cheer | Vocalic | Baseline/maintenance only |
| year | Vocalic | Baseline/maintenance only |
| sir | Vocalic | Baseline/maintenance only |
| stir | Vocalic | Baseline/maintenance only |
| butter | Vocalic | Baseline/maintenance only |
| flower | Vocalic | Baseline/maintenance only |
| mother | Vocalic | Baseline/maintenance only |
