# Supplementary material for: Differential Effects of Visual-Acoustic Biofeedback Intervention for Residual Speech Errors
Source: Front Hum Neurosci. 2016 Nov 11;10:567. doi: 10.3389/fnhum.2016.00567 (PMC5104733; doi:10.3389/fnhum.2016.00567)
Supplement: Supplementary file 2 [file Data_Sheet_2.DOCX]

**Appendix B. Details of Protocol for Online Collection of Perceptual Ratings**

Binary perceptual ratings of rhotic sounds elicited in probe measures were collected from non-specialist listeners recruited through the online crowdsourcing platform Amazon Mechanical Turk (AMT) and directed to a task hosted on the Experigen online experiment presentation platform (Becker & Levin, 2010). Following McAllister Byun, Halpin, & Szeredi (2015), each token was initially presented to nine unique listeners for rating. Due to data loss, such as cases in which a sound file failed to play, fewer than nine responses were collected for a subset of items. Items rated by eight unique listeners were considered adequate for inclusion in the analysis; items with seven or fewer ratings were recycled in clean-up blocks in order to collect additional ratings. If at least eight ratings had not been collected after three clean-up rounds, items were discarded.

Upon initiating the task, raters were informed that they would hear words containing “r sounds” produced by children of varying ages, and that their job was to rate each “r sound” as correct or incorrect. In each trial, participants saw the target word in standard orthography and heard the child’s production of the word, which they could listen to up to three times. Prior to rating any experimental stimuli, raters were required to complete 20 training trials in which they received feedback on the accuracy of their responses. Raters were then required to complete a 100-item eligibility test, in which their responses were evaluated using a criterion that combines acoustic measures and experienced listener ratings, as described in McAllister Byun, Halpin, & Szeredi (2015). Only raters who passed this criterion were eligible to proceed to rate experimental trials for pay.

Files were presented in random order in blocks of 220, of which 150 were experimental trials, 50 were filler trials, and 20 were catch trials. Half of the filler files were collected from typically developing speakers, and half were elicited from children receiving treatment in a previous study. Fillers were included so that the sample of words raters heard would not be overly skewed toward incorrect productions, and so that raters would not become overly familiar with the voices of the seven participants in the present study. The 20 catch trials were items hand-selected to represent unambiguously correct or incorrect production. They were used to monitor a rater’s attention to the task: if a participant did not score above chance on the catch trials in a block, the rater received a warning and results from that block were discarded. Raters could complete multiple blocks, but after five blocks, they were required to pass the eligibility test again in order to continue
